# Impact of PCBM as a Third Component on Optical and Electrical Properties in Ternary Organic Blends

**DOI:** 10.3390/polym16101324

**Published:** 2024-05-08

**Authors:** Laura Hrostea, Anda Oajdea, Liviu Leontie

**Affiliations:** 1 Research Center on Advanced Materials and Technologies (RAMTECH), Department of Exact and Natural Sciences, Institute of Interdisciplinary Research, Alexandru Ioan Cuza University of Iasi, 11 bd. Carol I, 700506 Iasi, Romania; 2 Faculty of Physics, Alexandru Ioan Cuza University of Iasi, 11 bd. Carol I, 700506 Iasi, Romania; anda.oajdea@gmail.com

**Keywords:** ternary organic films, fullerenes, constituents’ compatibility, electrical mobility, CELIV method

## Abstract

This paper investigates the influence of constituent weight ratios on optical and electrical properties, with a particular focus on the intrinsic properties (such as electrical mobility) of ternary organic blends, highlighting the role of a third component. The study explores novel donor:acceptor1:acceptor2 (D:A_1_:A_2_) matrix blends with photovoltaic potential, systematically adjusting the ratio of the two acceptors in the mixtures, while keeping constant the donor:acceptor weight ratio (D:A = 1:1.4). Herein, depending on this adjustment, six different samples of 100–400 nm thickness are methodically characterized. Optical analysis demonstrates the spectral complementarity of the component materials and exposes the optimal weight ratio (D:A_1_:A_2_ = 1:1:0.4) for the highest optical absorption coefficient. Atomic force microscopy (AFM) analysis reveals improved and superior morphological attributes with the addition of the third component (fullerene). In terms of the electrical mobility of charge carriers, this study finds that the sample in which A_1_ = A_2_ has the greatest recorded value [$\mu_{max}=1.41\times 10^{-4}{\mathrm{cm}}^{2}/(\mathrm{Vs}$)]. This thorough study on ternary organic blends reveals the crucial relationship between acceptor ratios and the properties of the final blend, highlighting the critical function of the third component in influencing the intrinsic factors such as electrical mobility, offering valuable insights for the optimization of ternary organic solar cells.

## 1. Introduction

In the context of the worldwide energy crisis and the issues arising from the greenhouse effect, renewable energy sources are gradually overtaking traditional sources of energy [1,2,3,4]. The sun is the most significant and cleanest energy resource since it is a limitless source of free fuel that has the enormous potential to supply much more energy than the planet needs. Nevertheless, there are certain restrictions on how photovoltaic devices (solar cells) can absorb solar energy and convert it into electricity [5]. The most crucial requirements for solar cells are a high photon-to-electron conversion efficiency and affordable production costs, adding to these the lifetime, stability, availability, and performance of the materials on which they are based [6]. Although first- and second-generation silicon solar cells have shown themselves to be a successful evolving photovoltaic technology, their current high costs restrict them from being used widely around the world. As a result of this, the development of photovoltaics has progressed along two paths: either silicon is substituted with other materials, or silicon is used in various solar cell architectures (such as silicon-based tandem solar cells and perovskite solar cells) [7]. In contrast, third-generation photovoltaics (OSCs—organic solar cells), based on organic materials, have shown notable scientific and technological progress [8].

In organic solar cells, the development of acceptor materials can be distinguished by comparing fullerenes with the more modern non-fullerene acceptors (NFAs) [8,9]. Although fullerenes have benefits, such as strong electron withdrawal and good miscibility with donor polymers, they also have disadvantages, including poor light absorption and challenging chemical tuning. However, because of their wide absorption range, simplicity in synthesis, and chemical tunability, NFAs show great promise. Furthermore, this field’s research recognizes commensurate developments in donor polymers, emphasizing the application of chlorinated conjugated polymers for accurate molecular-level modifications [10,11].

An approach to improving the photovoltaic performance of solar cell devices consists of the development of ternary organic solar cells (TOSCs) [12], which exploit the synergistic effects of tandem solar cells with those of classical bulk-heterojunction solar cells—all-in-one. TOSCs are based on an active layer consisting of the classical donor/acceptor (D/A) matrix that incorporates, as a third element, an electron donor or an electron acceptor material [13,14]. This approach gives the opportunity of choosing materials with the most promising photovoltaic potential and complementary features [15,16], while the role of the third component is crucial, as it supports an expanded optical absorption spectrum and facilitates the charge carrier transport, demonstrated in previous papers [17]. More than that, improvements to the polymers’ chemical structures, which serve as donor materials, can be carried out concomitantly [18,19]. Another important advantage, besides the photovoltaic potential improvement, is the simplicity and the ease of fabricating this type of cell, requiring only a combination of straightforward steps similar to those used in bi-layered cells fabrication. The latest (2024) record efficiency of a ternary organic solar cell is 19.13% [20].

Knowledge about the transport mechanism in ternary organic blends helps in understanding and developing further photovoltaic devices, given that charge carrier transit may limit solar energy conversion. Experimentally, a precise charge transport model in multi-component bulk heterojunction films has not yet been fully developed, despite the existence of some empirical criteria for mobility evaluation [21]. Thus, four different models are proposed to suitably describe the transport mechanism. Based on the energy properties and matrix type, they can be used to explain the basic mechanism of ternary cells. The charge transfer model, the parallel model, the alloy model, and the energy transfer model are the corresponding models [14].

Since charge carrier mobility is one of the inherent difficulties, assessing it might help identify any constraints or potential challenges to raising the efficiency of photon-to-electron conversion. Due to these key details, a number of methods for measuring charge carrier mobility have been created. The well-known method for measuring electrical mobility, presented by G. Juska in 2000 [22], called Charge Extraction by Linearly Increasing Voltage (CELIV), still has a number of advantages over other methods, the including Space Charge Limited Current (SCLC) and Time-of-Flight (TOF) techniques. One significant benefit is the ability to investigate multiple factors at once, including charge carrier mobility, relaxation time, and charge density. A triangle voltage pulse is applied to the blocking electrode (anode) of a sample structure in order to evaluate the equilibrium charge carrier current transients. As a result, the applied voltage pulse will move the equilibrium charge carriers to the opposite electrode (cathode, which is an ITO electrode connected to the negative terminal), where they will be extracted [23,24,25,26,27,28,29].

The novelty of this study is in how the adjustment of the constituents’ weight ratio in a ternary organic blend, especially the presence of the third component, impacts the electrical mobility and other significant intrinsic parameters. New emergent donor:acceptor1:acceptor2 matrix blends for ternary solar cell applications, based on a polymer (PBDB-T-2Cl), a non-fullerene (ITIC-F), and a fullerene (PCBM), are a main focus of this study.

## 2. Materials and Methods

The samples include blended constituent materials, separately bought from Sigma Aldrich (Steinheim, Germany): a conjugated polymer, PBDB-T-2Cl (Poly[(2,6-(4,8-bis(5-(2-ethylhexyl-3-chloro) thiophen-2-yl)-benzo[1,2-b:4,5-b’]dithiophene))-alt-(5,5-(1’,3’-di-2-thienyl-5’,7’-is(2ethyl hexyl)benzo[1’,2’-c:4’,5’-c’] dithiophene-4,8-dione)]), a non-fullerene ITIC–F (3,9-bis(2-methylene-((3-(1,1-dicyanomethylene)-6,7-difluoro)-indanone))-5,5,11,11tetrakis(4-exyl phenyl)-dithieno[2,3d:2’,3’-d’]-sindaceno[1,2-b:5,6-b’]dithiophene), and a fullerene, ([6,6]-Phenyl-C61-butyric acid methyl ester).

**Figure 1:**
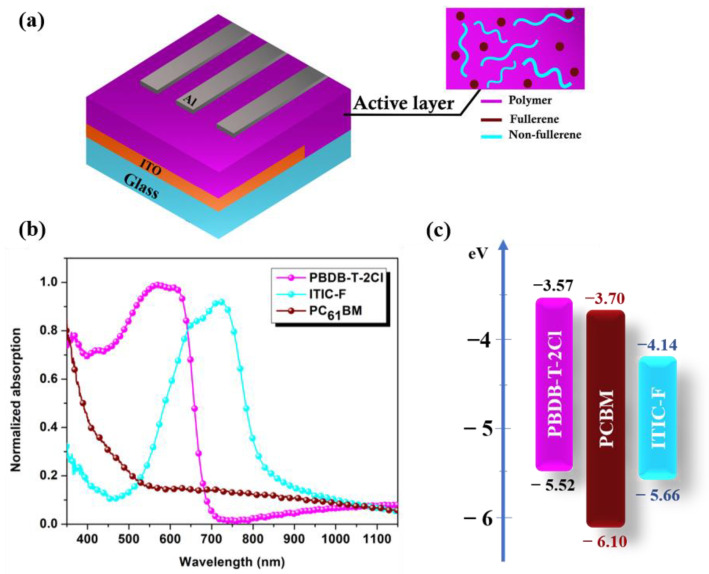
Schematic device structure (**a**); normalized absorption spectra of neat PBDB-T-2Cl, ITIC-F and PCBM thin films (**b**); and energy levels diagram of constituent materials (**c**).

Both Figure 1a,b show the schematic device construction (not to scale) and the optical characteristics of the neat constituents (polymer, non-fullerene, and fullerene as individual thin films). Furthermore, Figure 1c describes the molecular orbital energy levels (HOMO and LUMO) [30].

Chlorobenzene was used as a solvent. In advance, a mixture containing chlorobenzene (CB) and 1,8-diiodooctane (DIO) with a concentration of 3% was prepared. The first step was to dissolve the total amount of polymer in the prepared solvent (CB:DIO). This solution was magnetically stirred for 1.5 h at a temperature of 50 °C. Subsequently, five mixtures of 20 mg/mL concentration were prepared using the initial polymer solution, varying the acceptors’ weight ratio, labelled as [sample name = polymer:non-fullerene:fullerene]: #S1 = 1:0:0; #S2 = 1:1.4:0; #S3 = 1:1:0.4; #S4 = 1:0.7:0.7; #S5 = 1:0.4:1; #S6 = 1:0:1.4. The substrates were cleaned for 10 min in an ultrasonic bath with acetone, ethanol and detergent and the solutions were spun coated as thin films (100–400 nm thickness as presented in Table 1) at 1500 rpm spinning speed. Samples were dried at 100 °C for 10 min after deposition. The entire preparation process was carried out in a clean room under a lab hood that provided a normal/constant environment, and samples were kept in the dark. The aluminum digital cathode was deposited by thermal evaporation in a vacuum.

A DektakXT Stylus profilometer (Bruker France S.A.S., Wissembourg, France) was utilized to determine the thickness of the samples. A TEC5 spectrophotometer was used for the analysis of the optical absorption. Using an NT-MDT Solver Pro-M system (from NT-MDT, Moscow, Russia), atomic force microscopy (AFM) pictures were acquired. The measurements were performed using a SiN cantilever (NSC21 from Mikromasch, Tallinn, Estonia) in non-contact mode at room temperature. Using Nova software (version 1.0.26.1443) from NT-MDT, the samples’ root mean square roughness (RRMS) was calculated for a 1.5 × 1.5 µm^2^ scanned area. The XPS analysis was performed on a Physical Electronics PHI 5000 VersaProbe instrument (Ulvac-PHI, Inc., Chikasaki, Japan), equipped with a monochromatic AlKα X-ray source (hv = 1486.6 eV). The take-off angle of the photoelectrons was equal to 45°. All the XPS peak positions in the survey spectra were calibrated with respect to the C 1 s peak−binding energy (BE) = 284.6 eV. The CELIV (charge extraction by linearly increasing voltage) method was used to determinate the electrical mobility, wherein a triangular-shaped bias voltage pulse was applied from a AFG31022 function generator and the extracted current transient’s signal was recorded by a digital oscilloscope. The analysis involved changing the applied voltage from 1 V to 10 V while maintaining a consistent signal period of 30 μs. Under normal lighting conditions, several heating and cooling cycles were applied during the electrical measurements, which were performed in a perpendicular geometry configuration. The temperatures ranged from 30 °C to 120 °C.

## 3. Results and Discussions

**Figure 2:**
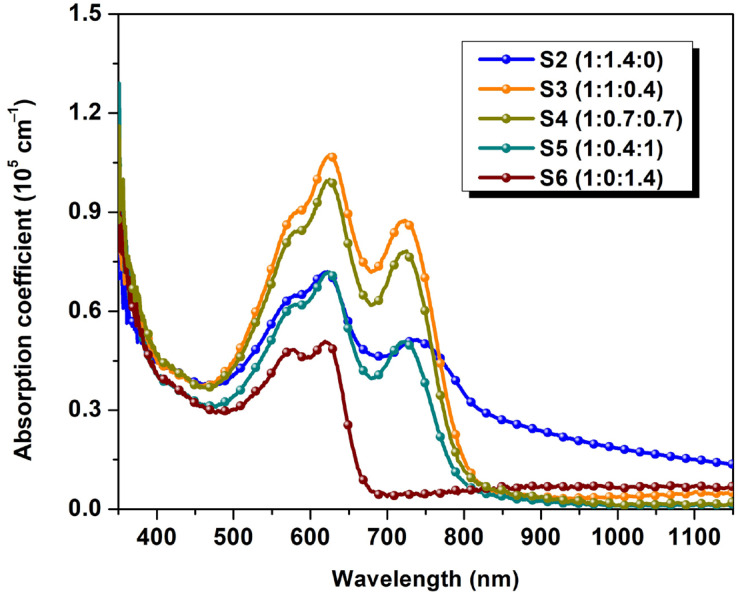
Absorption coefficient spectra of binary and ternary blends thin films.

Figure 1b shows the optical absorption spectra (normalized to 1) of the components in the blends. The preferred photon-harvesting wavelength ranges of each of the constituent materials, deposited as individual material thin films, are as follows: PBDB-T-2Cl thin film reveals an absorption band within the visible range between 450 and 700 nm, ITIC-F thin film displays a red-shifted extended band ranging between 500 and 850 nm with a maximum peak at 760 nm, and, in comparison to the non-fullerene and polymer components’ properties, PCBM thin film shows a notable absorption near the ultraviolet wavelengths, up to 400 nm, with a smaller absorption peak. Furthermore, the absorption of photons with wavelengths longer than 500 nm by PCBM continues to decrease towards the infrared region [31]. Because of the optical absorption complementarity of these materials, the photon harvesting of ternary blend thin films is increased and extended, as seen in Figure 2. As a result, NFA-based (binary and ternary) blends have broadened absorption spectra ranging from 500 to 800 nm, with two peaks indicating polymer and NFA fingerprints’ involvement. Small adjustments in the quantity of the third element can result in significant changes to the dielectric constant. The dielectric environment changes as acceptor elements are introduced into the polymeric matrix, enhancing its character, as previously reported in [32]. Considering solely ternary blends, the absorption coefficient decreases as the amount of NFA in the blends diminishes. Sample #S3 (1:1:0.4) has the highest absorption coefficient and the longest wavelength as absorption edge (Table 1), considering the fact that the optical absorption edge shifts to lower energies (longer wavelengths) when the non-fullerene is present in the matrix.

**Figure 3:**
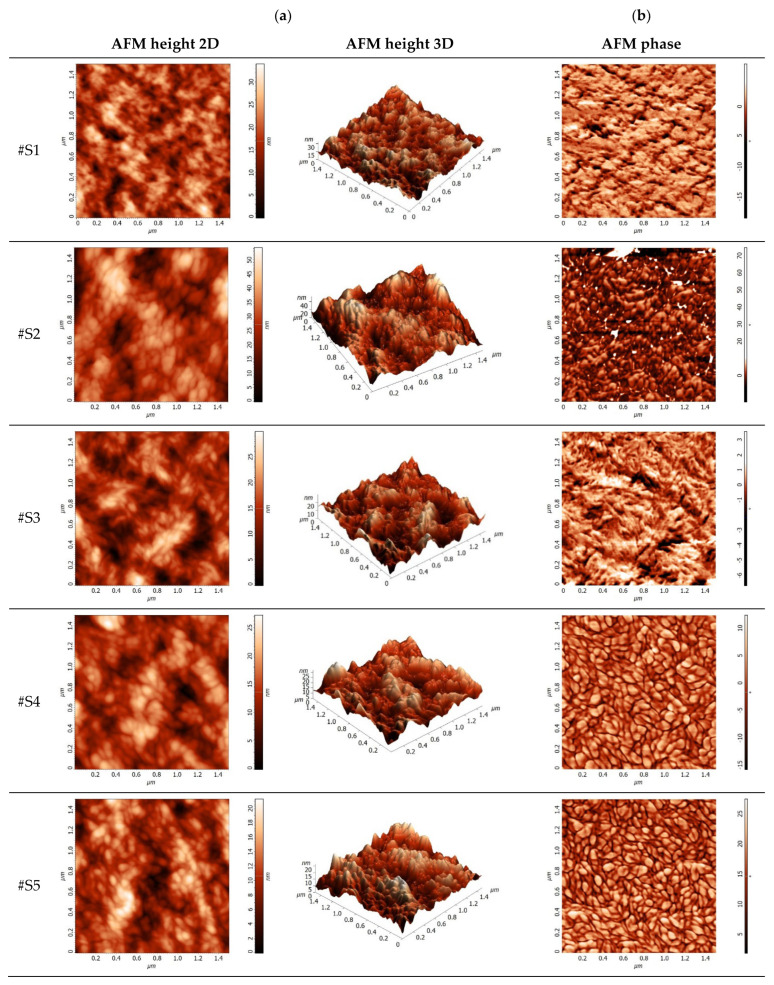

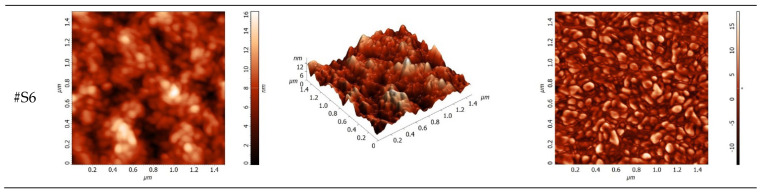
AFM analysis: height images in 2D and 3D representation (**a**) and phase images (**b**).

Thus, the AFM height images (2D and 3D—Figure 3a) and the AFM phase images (Figure 3b) illustrate a key characteristic of the samples. The AFM height images describe the samples by determining the roughness. According to Table 1, the nanoscale smooth topography of the samples is observed, where the specific root mean square roughness (R_RMS_) of the polymer thin film (#S1) is 4.97 nm. By adding the non-fullerenes, this value increases to 8.24 nm and then gradually moderates with the addition of fullerene. This is due to a higher miscibility of the fullerenes and non-fullerenes in the polymer matrix. Good miscibility between materials reduces the driving force for phase separation, which results in smaller impurity domains, Figure 3b, that support efficient electron-hole dissociation [31,33]. Higher-performance OSCs require compatible and favorable film morphology in the active layer. The ternary blend thin films exhibit smoother topography compared to polymer (#S1) or fullerene-free thin films (#S2), confirming the enhanced miscibility of the three constituent materials and remarking on the crucial role of the third component—PCBM, which can be considered a morphology regulator in adjusting the molecular arrangement of the polymer:non-fullerene host matrix [34]. When the amount of fullerene in the polymer:non-fullerene host matrix is raised, it is evident, from comparing the AFM phase images of all the samples, that highly well-defined and shaped nanodomains are revealed. This fact is based on PCBM’s higher tendency to form clusters, which favors the development of nanodomains. In contrast to fullerene-free blends, the composition differences between fullerene domains, non-fullerene domains, and the polymer matrix become more prominent at increasing PCBM concentrations, providing a stronger contrast in AFM phase images of more easily discernible nanodomains [35].

**Figure 4:**
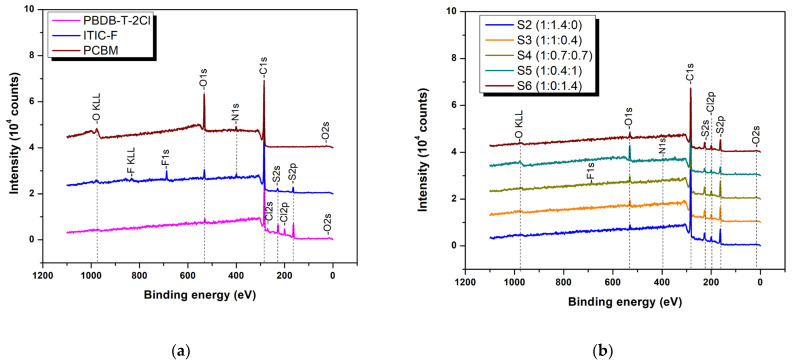
XPS survey spectra of individual material thin films coated on glass (**a**) and binary and ternary organic thin films (**b**).

A well-mixed material surface is revealed by the XPS survey spectra, Figure 4, which displays the surface compositional profiles down to around 10 nm in depth. Even though the third component, PCBM, is hydrophilic, this surface composition is consistent with [17], which emphasizes the hydrophobic nature of the layer surface even though it may not accurately reflect the distribution of elements within the complete layers. According to this information, PCBM is anticipated to settle in the region between the polymer and non-fullerene host matrix. In addition, certain non-fullerene’s atoms, such the fluor atom, are likely to be highlighted at the surface of thin films based on binary and ternary blends. As a result, this does suggest that the polymer matrix contains the two integrated acceptor materials, fullerene and non-fullerene. Together with the XPS data, [34] also confirms the compatibility and good miscibility of the PCBM and ITIC-F materials, which are incorporated in the polymeric matrix to form an effective electron transport network.

**Figure 5:**
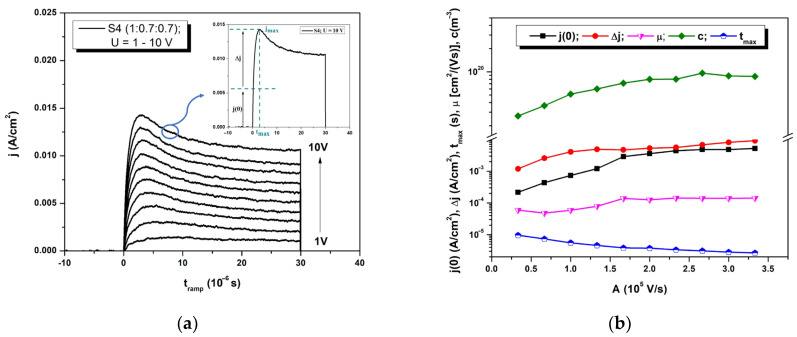
Analyze on sample S4: CELIV signal variation with the applied voltage (**a**); intrinsic parameters determined from the current transients (**b**).

To analyze the intrinsic parameters, such as charge carrier mobility, the CELIV method is widely used for determining the bulk transport properties of materials in sandwich geometry, especially in disordered materials such as organics [36,37,38]. Regarding the transport mechanism, the parallel model provides the best description of the transport process in the case of the investigated D:A_1_:A_2_ matrix, where the active layer functions as two intercalated bilayers (D:A_1_ and D:A_2_) [17]. Due to the third element’s function as an electron transfer channel, the compounds’ compatibility and miscibility are crucial. In this case, holes are created in the D domains as electrons move to the nanoscale domains of A_1_ and A_2_. Therefore, knowing that the investigated samples are based on donor:acceptor blends of a 1:1.4 weight ratio, this suggests that since the acceptor concentration is higher, the positive charge carriers (holes) are the majority carriers. The method is based on investigation of the extraction of equilibrium carries, in which a linearly increasing voltage is applied. Hence, by applying the voltage [$U\left(t\right)=A\cdot t$, where *A* is the voltage ramp], a current transient signal is obtained, as shown in Figure 5a. Charge injection is avoided due to the presence of a blocking contact from the sandwich-like structure of the device. The total current transient signal is composed from *j*(0), the initial current step, caused by the geometrical capacity of the sample, giving information about the material permittivity or about the interelectrode distance [$j\left(0\right)=\frac{\varepsilon \varepsilon_{0}A}{d}$], and Δ*j*, caused by the electrical conductivity of the sample [ $\sigma =\frac{3\varepsilon \varepsilon_{0}\Delta j}{2t_{max}j\left(0\right)}$], which is the supplementary current formed by the extracted charge carrier. The total current transient, *j_max_*, reaches a maximum value at *t_max_*, as is exemplified in Figure 5a inset. By applying Formula (1), knowing the thickness *d*, the mobility of the faster charges can be determined:

(1) $$\mu =\frac{2}{3}\frac{2d^{2}}{At_{max}^{2}\left[1+0.36\left(\frac{\Delta j}{j\left(0\right)}\right)\right]}$$

The electric-field dependence of the intrinsic behavior of the charge carrier was determined by varying the maximum of a triangular voltage pulse between 1 and 10 V, and the current transient signals can be seen in Figure 5a for one sample, #S4.

Herein, the current transient increases rapidly until the majority of charge carriers have been extracted at *t_max_*, at which point it sharply declines [28]. Compared to other methods, CELIV is based on the extraction of equilibrium charge carriers (shallow trapped) and, increasing the voltage, the extracted carriers are located even on deeper states, leading to a prominent CELIV current [39]. More than that, the evolution of some parameters is presented in Figure 5b.

Using the analyses of sample #S4 as an example, it is noticeable that the increase of the voltage exerts an influence on the transport of charge carriers within the thin film (Table 2). In this context, the mobility exhibits a proportional increase with the applied voltage, attaining its maximum value at $\mu_{10V}=1.41\times 10^{-4}{\mathrm{cm}}^{2}/\mathrm{Vs}$, corresponding to the values reported in [31] or even higher [40]. This fact is based on Poole–Frenkel model, consisting of an increased probability of electric-field-stimulated charge carrier release from localized states [41]. Conversely, there is an anticipated inverse correlation observed in the time required for the current to reach its peak value [42], denoted as $t_{max}=2.66\times 10^{-6}\;s$. *T_max_* increases as the speed of the voltage rise *A* decreases, indicating the electrical field dependence of mobility [42]. The concentration of extracted charge carriers ($c\sim 10^{20}\;m^{-3}$) is estimated, calculated from the area under the extraction peak. As inferred from the current transient components [*j*(0) and Δ*j*], it also manifests an elevation concomitant with the incremental voltage ramp. 

Acceptors may affect the morphology of the blend inside the layer, as the AFM images demonstrate. For hole transfer, a well-defined interconnected network is essential. The ideal ratio of constituents can be found by adjusting the weight ratio of the acceptors, which will improve the packing of the donor polymer while making hole hopping between donor molecules easier. Furthermore, the effectiveness of charge hopping depends on the energy levels of the donor polymer and both acceptors. The right weight ratio minimizes the energy barrier for charge transport and facilitates effective transfer from the donor to the acceptors. By adding more intermolecular interactions to the blend, the PCBM acceptor acting as third component has an impact on mobility and the electronic coupling between donor molecules.

**Figure 6:**
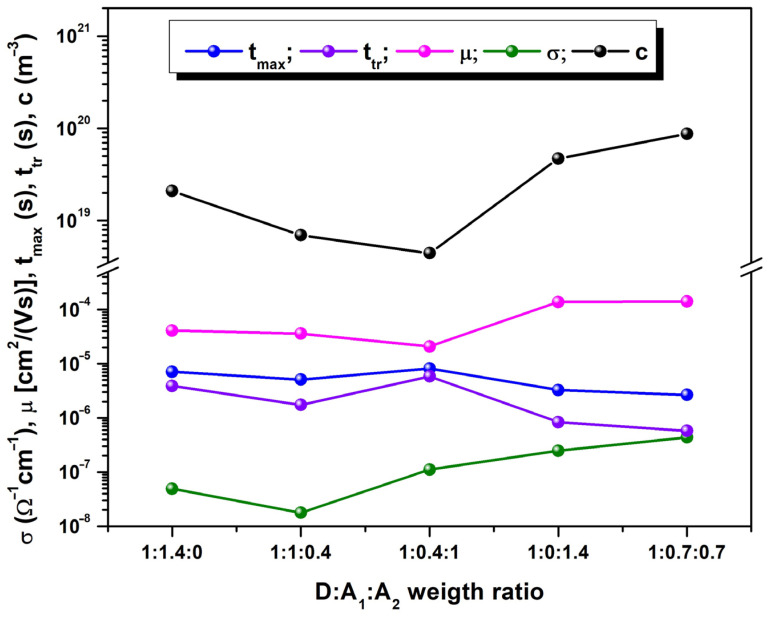
CELIV parameters of samples depending on the weight ratio variation.

Despite maintaining a constant donor:acceptor ratio across all the blends of thin films (D:A = 1:1.4), the variation in the weight ratio of the acceptors (A_1_:A_2_) is observed to impact charge carrier transport, as illustrated in Figure 6. Notably, the electrical conductivity experiences an increase with the augmentation of fullerene content in the blend, a fact confirmed by four-probe method assessment, wherein the highest conductivity value is $\sigma_{S4}=4.4\times 10^{-7}\Omega^{-1}{\mathrm{cm}}^{-1}$, corresponding to sample #S4. Additionally, in terms of mobility, sample #S4 (wherein A_1_:A_2_ = 1) attains the highest value [$\mu_{max}=1.41\times 10^{-4\;}{\mathrm{cm}}^{2}/\left(\mathrm{Vs}\right)]$, at U=10 V, followed by sample #S5, wherein the non-fullerene component is absent. Once the fullerene amount decreases, the electrical mobility registers lower values. Consequently, both *t_max_* and *t_tr_* (carrier transit time of interelectrode distance) reach their minimal values for the same blend ($t_{max}^{S4}=2.66\;\mu s$ and $t_{tr}^{S4}=0.57\;\mu s$).

## 4. Conclusions

The present study into donor:acceptor1:acceptor2 (D:A_1_:A_2_ = PBDB-T-2Cl:ITIC-F:PCBM) ternary organic blends demonstrates the significant impact of varying the acceptors’ weight ratio on their optical, morphological, and electrical properties. Sample #S3, with a weight ratio $A_{1}>A_{2}$ and an absorption maximum $\alpha =1.07\times 10^{5}{\mathrm{cm}}^{-1}$ at 628 nm wavelength, has superior optical properties. AFM characterization shows a very smooth nanoscale topography, remarking that a decrease in roughness is correlated with an increase in fullerene content in the blend. This fact indicates that fullerenes function as binders in the polymer-fullerene matrix. The electrical properties are also improved as the amount of fullerene in the blends increases; sample #S4 (A_1_ = A_2_) exhibits the highest electrical conductivity ($\sigma_{S4}=4.4\times 10^{-7}\Omega^{-1}{\mathrm{cm}}^{-1}$) and charge carrier mobility [$\mu_{S4}=1.41\times 10^{-4\;}{\mathrm{cm}}^{2}/(\mathrm{Vs}$)]. This study also reveals the electric-field dependence of intrinsic parameters, determined using the CELIV method, and the variability of the applied voltage shows significant changes in the transient current density, highlighted by #S4 analysis. These results highlight how important it is to take into consideration the trade-off between optical, morphological, and electrical properties when designing ternary blends suitable for photovoltaic applications. This work shows that electrical mobility and other intrinsic properties are significantly influenced by varying the weight ratio of acceptors, specifically the impact of the third component. This study offers perspectives for developing highly efficient organic solar cells with an optimized structure.

## Figures and Tables

**Table 1:** Thickness and roughness values of investigated samples.

Sample	D:A_1_:A_2_ Weight Ratio	Thickness (nm)	Absorption Edge (nm)	R_RMS_ (nm)
S1	1:0:0	100	656	4.97
S2	1:1.4:0	400	685	8.24
S3	1:1:0.4	250	780	4.65
S4	1:0.7:0.7	285	775	3.91
S5	1:0.4:1	350	775	3.37
S6	1:0:1.4	210	656	2.42

**Table 2:** Determined parameters compared to the literature.

Parameters	Maximum Determined Values	Literature Reported Values	
$\mu_{max}$	1.41 × 10^−4^ cm^2^/Vs	1 ÷ 6 × 10^−5^ cm^2^/Vs	[40]
*c*	10^20^ m^−3^	4.73 × 10^20^ m^−3^	[43]
*t_tr_*	0.57 µs	0.55 ÷ 0.70 µs	[44]
*α*	10^5^ cm^−1^	3.5 × 10^4^ cm^−1^	[45]

## Data Availability

Data are contained within the article.
